# Supplementary material for: Association between Dietary Habits and Severity of Symptoms in Premenstrual Syndrome
Source: Int J Environ Res Public Health. 2023 Jan 17;20(3):1717. doi: 10.3390/ijerph20031717 (PMC9914022; doi:10.3390/ijerph20031717)
Supplement: Supplementary file 1 [file ijerph-20-01717-s001.zip › ijerph-2175541-supplementary.pdf]

Table S1. Questionnaire used in the study to record data of participating women.

Date	(d/m/y)
Date of Birth	(d/m/y)
Weight	(Kg)
Height	(cm)
Age of menarche	(Years, months)
Do you have regular menstrual cycles?	Yes/No
Days between menstruations (average)	
Do you know to have Premenstrual Syndrome?	Yes/No
What is the average length of your menstrual flow?	(days)
Do you regularly consume drugs?	Yes/No
- If yes, can you specify?	
Are you on EP pill?	Yes/No
Do you regularly exercise (at least three times per week for one hour)	Yes/No
- Please specify	
Do you consume coffee?	Yes/No
- How many cups/day	

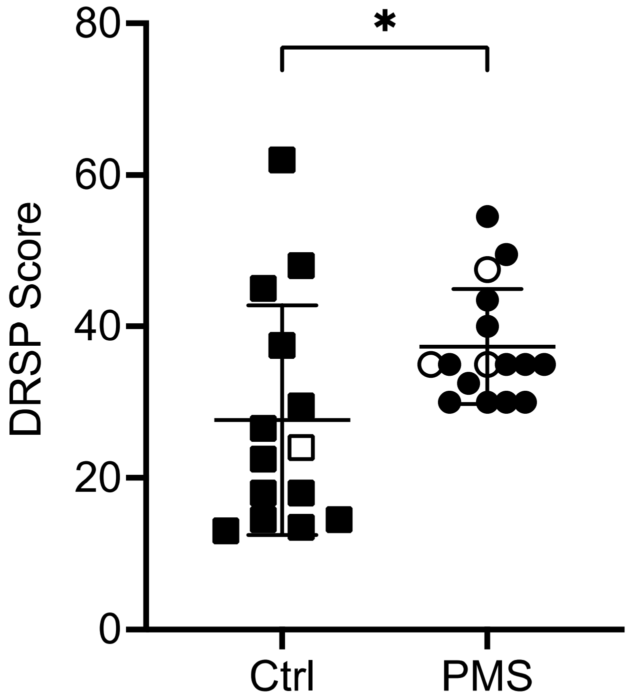

Figure S1. DRSP scores of participating women. Squares represent normal women, circles represent women diagnosed with PMS according to [24]. hite symbols represent participants taking the pill EP. Significance was calculated using the Mann-Whitney test. * p = 0.0142.

Figure S2.

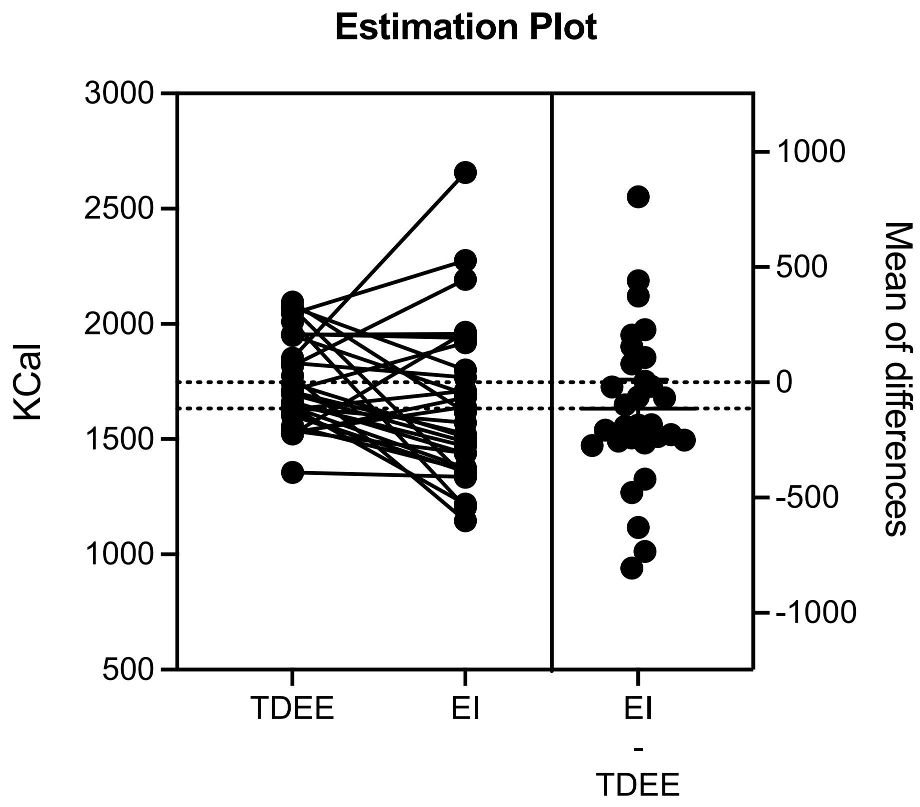

Figure S2. Comparison between estimated total daily energy expenditure (TDEE) and energy intake (EI) calculated from food diaries. TDEE and caloric intake calculated using Student's t-test for paired data were not significantly different (p = 0.0751).
